# Impact of COVID-19 on the Mental Health of Children and Adolescents

**DOI:** 10.7759/cureus.10051

**Published:** 2020-08-26

**Authors:** Kaushal Shah, Shivraj Mann, Romil Singh, Rahul Bangar, Ritu Kulkarni

**Affiliations:** 1 Psychiatry, Griffin Memorial Hospital, Norman, USA; 2 Research, All Saints University School of Medicine, Roseau, DMA; 3 Internal Medicine, Metropolitan Hospital, Jaipur, IND; 4 Internal Medicine, Adesh Institute of Medical Sciences and Research, Bathinda, IND; 5 Medicine, University of Oklahoma, Norman, USA

**Keywords:** covid-19, child and adolescent psychiatry, psychological first aid, school psychology, quarantine, online education, school counseling

## Abstract

The coronavirus disease 2019 (COVID-19) outbreak was first reported in Wuhan, China, and was later reported to have spread throughout the world to create a global pandemic. As of August 18th, 2020, the coronavirus had spread to more than 216 countries with at least 21,756,357 confirmed cases, resulting in 771,635 deaths globally. Several countries declared this pandemic as a national emergency, forcing millions of people to go into lockdown. This unexpected imposed social isolation has caused enormous disruption of daily routines for the global community, especially children. Among the measures intended to reduce the spread of the virus, most schools closed, canceled classes, and moved it to home-based or online learning to encourage and adhere to social distancing guidelines. Education and learnings of 67.6% of students are impacted globally due to coronavirus in 143 countries. The transition away from physical classes has significantly disrupted the lives of students and their families, posing a potential risk to the mental well-being of children. An abrupt change in the learning environment and limited social interactions and activities posed an unusual situation for children's developing brains. It is essential and obligatory for the scientific community and healthcare workers to assess and analyze the psychological impact caused by the coronavirus pandemic on children and adolescents, as several mental health disorders begin during childhood. Countries across the globe, including the United States, are in the dilemma of determining appropriate strategies for children to minimize the psychological impact of coronavirus. The design of this review is to investigate and identify the risk factors to mental health and propose possible solutions to avoid the detrimental consequence of this crisis on the psychology of our future adult generations.

## Introduction and background

Since the first reported coronavirus case in Wuhan, China, in 2019, the outbreak, now known as COVID-19, has spread globally [1]. The World Health Organization (WHO) acknowledged this coronavirus epidemic as a pandemic and declared the outbreak as a public health emergency of international concern [2-3]. Most regions around the world are affected severely, including the United States, Brazil, India, Russia, and Europe, which have seen an increasing number of cases and deaths than the rest of the world [3-4]. As of August 18th, 2020, the coronavirus had spread to more than 216 countries and has at least 21,756,357 confirmed cases, resulting in 771,635 confirmed deaths globally. In the United States, between January 20th and August 18th, 2020, there have been 5,354,013 confirmed cases of COVID-19 with 168,999 deaths [5]. The spread of the virus has caused global economic and social disruptions and has brutally overwhelmed the healthcare and educational systems [6]. 

The unexpected disruption of the social fabric and norms has affected the behavioral and mental health of the public, including children. The mental health of children has been influenced by several ways, as this unprecedented situation changed a way they typically grow, learn, play, behave, interact, and manage emotions. Children with pre-existing psychiatric disorders such as attention-deficit/hyperactivity disorder (ADHD), anxiety, depression, mood disorders, and behavior disorders could be adversely impacted during this stressful situation [7]. Mental disorders are the leading cause of disability worldwide in adolescents and children. About 15% of children and adolescents in the world have mental health disorders or conditions. Nearly 50% of mental disorders start to affect the children by the age of 14. If left untreated, a child's mental development has been found to be drastically and detrimentally impacted. It is well established that mental health is one of the essential parts of human development and determines the outcome of a child's educational attainments and the potential to live fulfilling and productive lives [8]. Mental illness can affect children at any point during their childhood, but it most significantly affects them during adolescence. Among the several mental illnesses that can be prevalent in childhood, depression is one of the major leading causes of mental illness amongst children. In 2016, an estimated 53,000 deaths were due to adolescent suicide, which is the third leading cause of morbidity in this group. This emphasizes that adolescence is a period of vulnerability for the onset of mental health conditions [9].

As of August 18th, 2020, 143 countries have closed schools and educational facilities worldwide due to the COVID-19 pandemic, impacting 1,184,126,508 learners, consisting of about 67.6% of students globally. It has forced several countries to implement home-based learning or online training [10-12]. Approximately 1.1 billion students and their families have been affected by school closures due to the pandemic. These students are experiencing further distress due to the unavailability of adequate help and attention from the trained instructors, making education more expensive for them and their families as they need to utilize additional time, support, and resources. Due to the closing of schools, students' interaction and communication with school mates, play, exercises, and peer- activities are hindered, which have proven vital for the growth, development, and learning of the young human minds [10]. The children who are at most significant risk are the youngest ones as their brains are still developing and are being exposed to high levels of stress and isolation, which can lead to permanent abnormal development. Children exposed to stressors such as separation through isolation from their families and friends, seeing or being aware of critically ill members affected with coronavirus, or the passing of loved ones or even thinking of their own death from the virus can cause them to develop anxiety, panic attacks, depression, and other mental illnesses [11-12]. 

The conducted literature search was through Medline, PubMed, PubMed Central, and Embase using the keywords, 'coronavirus,' 'COVID-19,' 'mental health,' 'child and adolescent,' 'behavioral impact,' 'psychological conditions,' 'quarantine,' and 'online education.' The indexed search aimed to identify literature and articles relevant to our focused topic. The objectives of this review article are 1. To understand the overall psychological impact of COVID-19 on children and adolescents; 2. Identifying factors contributing adversely to their mental health; and 3. Proposing interventions based on the guidelines and evidence-based practices.

## Review

Overview of mental health impact on children

The outbreak of COVID-19 has disrupted the lives of many people across the world. The pandemic has imposed a sense of uncertainty and anxiety, as the world was unable to predict or prepare for this crisis. It has caused a tremendous stress level among children, adolescents, and all students in general, primarily due to the closure of their schools. This stress may lead to undesirable adverse effects on the learning and psychological health of students [13]. Children exposed to these incidents can precipitate the development of anxiety, panic attacks, depression, mood disorders, and other mental illnesses [11]. Distressing events such as separation from family and friends, seeing or being aware of critically ill members affected with coronavirus, or the passing of loved ones or even thinking of themselves perhaps dying from the virus would have a detrimental effect on the mental health. Additionally, the healthy daily routines of children have been disrupted due to the COVID-19, which contributes to the additional stress and sleeping difficulties that many children face. Uncertainty of their future ambitions, academics, personal relationships, and inactivity due to the pandemic poses a significant threat to their mental well-being and putting them at risk of drug abuse [14]. COVID-19 can seriously leave a negative impact on children's mental health, just like other traumatic experiences humans may face. It can lead to higher rates of depression, anxiety, and post-traumatic stress disorder. This causes fear in children because the virus threatens not just them but also their families and surroundings, especially as they see their parents working from home, leading to fear and shock [15].

Evidence of psychological impact from the past outbreaks

Previous studies on severe acute respiratory syndrome (SARS), Middle East respiratory syndrome (MERS), and Ebola have revealed that the disease causes severe emotional distress during the outbreaks. Unfortunately, studies were not adequately conducted on the children and adolescents during the past outbreaks to measure its impact on their mental health, but several parallels can be drawn. The situation of COVID-19 is comparable with the MERS and SARS, as similar claims made about the severity of MERS caused fear, worry, and anxiety among the public. A study on the SARS survivors with psychiatric disorders revealed that about 25% of the patients showed signs of post-traumatic stress disorder (PTSD), and 15.6% of them had worsening depression [16]. This finding corresponds to the increased suicide deaths among SARS survivors, consisting of older adults from Hong Kong in 2003 and 2004 [17]. Among those MERS survivors, lower quality of life was also noticed. Neuropsychiatric linkage has been established based on the previous outbreaks [18].

Factors contributing to the mental health of children

During this pandemic, children and their families have been exposed to direct or indirect factors that could pose stress and emotional disturbance. Several weeks of homestay has forced parents and/or caregivers to work from home. Also, many families lost their financial independence due to job losses [11]. This disease is installing fear in children because children are worried about not only getting infected but also having their parents staying at home and not leaving for work [15]. Some families are struggling to feed their children, as many were dependent on school programs or food stamps, and not all families with resources can provide adequate supplies [19]. However, the reach of the pandemic is unequal as numerous families have lost loved ones while others live in regions untouched by the virus. Some children have parents who work on the front lines in COVID-19 settings, and others have parents who now work from home or have recently been terminated [19]. 

Additionally, international students are impacted by uncontrollable factors such as school closure, campus closure, and travel restrictions. Nations across the globe have restricted their borders to internationals to help mitigate the pandemic as many students might not have any other place to reside. This sudden closure of many nations to outsiders has placed a great burden on school administrators to ensure housing, sustenance, and safety of their international students [19-20]. While transitioning to online classes has helped both international and national students to continue their education, several children and faculty members are experiencing distress because they may not have the technological capability or expertise required to navigate this new mode of interaction. The online teaching method has raised questions for the faculty about their capability to deal with the existing technology [20]. The COVID-19 pandemic has caused unprecedented health and humanitarian crisis. It has created an economic downturn due to the necessary measures to contain the spread of the virus. As per the latest global financial stability report, there is likely to be financial instability, which would lead to a devastating recession. The combined economic uncertainty and emotional distress placed on a family will challenge the overall well-being of families as well as their mental health [21].

Interventions to mitigate psychological stressors

It is paramount to encourage and adopt healthy behavior to maintain the overall well-being of families. The well being of caregivers or parents can directly impact the mental health of the children. Parents are advised to follow and practice the guideline provided by the World Health Organization (WHO). The WHO has urged people to follow social distancing guidelines and avoid close contact with anyone, especially from the person showcasing any respiratory symptoms [22]. The health organization has also emphasized maintaining better hygiene by consistently washing hands and using appropriate protective gear such as facial masks [22]. It has also advised to take breaks from watching, reading, or listening to news stories, including social media, because continually being bombarded by news of the pandemic can be distressing. Exercising regularly, practicing yoga or meditation, eating healthy, taking adequate and proper sleeping properly, and avoiding alcohol or drugs is key to maintaining mental health. It is also crucial that parents provide enough support to their children and help them to process the information about the pandemic because these interventions could help minimize their anxiety or fear [22].

Schools, parents, and healthcare institutions can also implement psychological first aid (PFA) guidelines to assist children with their mental distress. PFA can provide psychosocial support to any survivors of epidemic or disaster [23]. It is developed to mitigate acute distress and assess the need for more advanced psychiatric care. It is beneficial to implement it during the early stages of crisis to assist survivors in coping with grief and avoiding the long-term impact of stress on mental health. The 'RAPID' model of the John Hopkins PFA tool includes five steps, (i) R - Rapport and reflective listening, implemented throughout the interaction; (ii) A - Assessing and evaluating the psychological needs; (iii) P - Prioritizing the needs based on severity; (iv) I - Intervening to mitigate distressing factors; (v) D - Disposition and distribution of intervention to stabilize the survivor [24-25].

Strategies for educational institutions

Schools should emphasize the mental health of students by supporting and providing updated health organization guidelines through online lectures. Also, a licensed counselor should help students manage the COVID-19 related stress by providing coping mechanisms and strategies in both group and individual sessions. Counseling services should be available to support the mental health and well being of students on time. Universities can establish a task force to make a plan to reduce the spread of the virus and for the following Centers for Disease Control and Preventions (CDC) guidelines. The committee should include members from diverse professional backgrounds and experiences, such as public health department, physicians, psychiatrists, psychologists, social workers, administrators, health and human services, international services center, human resources, admission offices, enrolment, and billing department, athletic department, and teachers. To reduce the distress experienced by students and faculty related to information technology (IT) issues, a technical team should be available continuously, and learning tutorial videos should be shared with the end-users. Similarly, teachers and faculty should support students and their parents through clear communication and assigning clear expectations [24]. 

A licensed counsel should take a comprehensive assessment of students deemed susceptible through risk factors such as psychological issues, including poor mental health before the crisis, bereavement, injury to self or family members, life-threatening circumstances, panic, separation from family, and low household income. Minimizing the interruption of psychiatric care for patients with pre-existing conditions via telepsychiatry will be helpful to continue monitoring patients as the pandemic may worsen some patients' conditions and would adversely impact them if they were unable to contact their doctor. Psychological assessment will help them to cope with their mental issues and stabilize their condition as they gain more education and discuss the impact of a pandemic. It will provide them support and reassurance to build resilience and encourage them to stay positive and motivated [26].

Evidence-based recommendations

Mental health involves the regulation of our emotions, psychological, and social well-being. Per the CDC, mental health affects how we think, feel, and act. It also helps determine how we react to stress, correlate with others, and our decision-making. Mental health is significant throughout our lives, from early childhood to adolescence and through adulthood. Mental illnesses occur when mental health is affected and leads to conditions that affect the way a person thinks, feels, or behaves, such as depression, anxiety, bipolar disorder, or schizophrenia. Mental health can cause conditions that may be acute or chronic and alter the way we live our lives daily by our rationalizations. Psychological and physical health are interdependent, both working together to form who we are. Mental illness, especially depression, limits rational thinking, and increases the risk for other health problems such as diabetes. The presence of chronic conditions can increase the risk of mental illness. It is vital to strike a healthy balance between students' physical and psychological well-being [26]. 

Protecting and maintaining the mental health of the future adult generation is only possible with the robust schooling and healthcare system. It is necessary to have adequate resources to overcome this crisis. Recruiting additional school personnel, clinicians, and mental health counselors are needed to address the strain on the system for supporting students during this pandemic [27-28]. Comprehensive school mental health systems (CSMHSs) is required to deliver adequate assistance for the students effectively [27]. CSMHSs is a school-community association developed for all students to provide a variety of services for every type of students, such as mental health services, health promotion and prevention, early identification and interventions of diseases, and treatments for students evidence-based medicine [27]. The CSMHSs should be enabled to collaborate with counselors, community mental health, and physical healthcare providers to help prevent mental health issues and make necessary referrals through an online interface for the treatment. The recruitment of additional school personnel and mental health counselors will help the students manage their anxiety, depression, and/or stress due to COVID-19; and to stabilize any previously diagnosed mental illness or prevent new mental illness from developing [27, 29]. 

Moreover, children with inadequate information about why quarantine measures have been taken are found to have more anxiety. Therefore, it is essential to expose children to more information about COVID-19 through several sources, such as the evening news [29-30]. This will make children more aware of the reason behind not only why quarantine measures were put in place, but they will also learn more about what COVID-19 is. Parents and guardians are encouraged to speak with their children about the information they learned, which may help lessen the negativity associated with COVID-19 and quarantine. Additionally, communicating with children about how they are processing the information will provide children with the emotional tools they require to do well in quarantine [27]. Not only can parents inform children about quarantine, but they can also employ "positive parenting" [31]. Children are prone to observe parents' and family members' moods during quarantine, which the children react to. Through positive parenting, parents, guardians, and family members can create consistent daily routines to avoid the distress of unstructured days [31-32]. 

While parents can provide a deeper understanding of the COVID-19 and quarantine, school systems can provide further reassurances and educate children about emotions [33]. School systems have the unique opportunity to provide consistent information to a large student body, who is unable to access other mental health programs in the areas [34]. Furthermore, school systems must adapt to the new online learning method and help students adjust and thrive in online classes [34-35].

Additionally, children can be taught coping mechanisms to self-regulate their own emotions without dependence on others. One method that achieves this goal is behavioral activation, which focuses on participating in activities they enjoy and not employing avoidance behaviors [31-32]. Alongside the other interventions mentioned above, behavioral activation can help children improve their problem-solving skills by engaging in healthy behaviors rather than unhealthy ones [27]. Due to the isolation indirectly imposed by the pandemic, children would be expected to prosper better in these times when they are taught ways to help themselves [31, 35].

## Conclusions

The overall understanding of COVID-19 has expanded throughout the world, yet its immediate and long-term mental health impacts on the children are challenging to estimate. Measures to prevent the virus from spreading and tackling the uncertain situations pose risks to the psychological well-being of the children. The steps taken, such as closing schools, limiting social interactions, imposing travel restrictions, halting sports activities, and transitioning all to online classes, have engendered emotional distress, fear, and anxiety amongst the children and their caregivers. It is essential that the guardians, educational institutions, and health authorities protect and guard the mental health of children consistently through open communication and facilitate professional counseling to address stressors. Additional attention should be given to the children who are more susceptible to the mental health crisis through a collaborative approach by involving their parents, educators, school administrators, counselors, psychologists, and psychiatrists.
